# From Pester Power to Purposeful Co-Shopping, a Dual-Pathway PLS-SEM Model: The Influence of Children’s Food Purchases in Urban India

**DOI:** 10.3390/bs16101768

**Published:** 2026-09-28

**Authors:** Murale Venugopalan, Preetha R., Gyanendra Singh Sisodia

**Affiliations:** 1 Amrita School of Business, Kochi Campus, Amrita Vishwa Vidyapeetham, Kochi 682 041, Kerala, India; v_murale@asb.kochi.amrita.edu; 2 Department of Commerce and Professional Studies, Rajagiri College of Social Sciences (Autonomous), Kalamassery 683 104, Kerala, India; preetha@rajagiri.edu; 3 College of Business Administration, Ajman University, Ajman P.O. Box 346, United Arab Emirates

**Keywords:** consumer socialization theory, reverse socialization, in-store promotions, peer-mediated influence, parent–child conflict, co-shopping, PLS-SEM, emerging markets

## Abstract

Children increasingly shape family purchasing decisions, not merely respond to them. Drawing on Consumer Socialization Theory, this study examines how in-store promotions affect children’s “pester power”, their persistence in asking parents to buy products directly and through peer influence. It also asks whether proactive parental engagement can redirect this pattern toward positive, collaborative shopping instead of conflict. Using PLS-SEM with survey data from 169 urban Indian parents of children aged 5–12, the study produces three findings. In-store promotions are the strongest market driver of both peer influence and pester power. Peer influence fully mediates advertising’s weaker, indirect effect on pester power, suggesting that market stimuli gain force mainly through social amplification among children’s peers rather than direct exposure alone. Pester power, in turn, strongly predicts parent–child conflict over food purchases. Third, and central to this study’s contribution, proactive parental engagement independently predicts positive co-shopping experiences, regardless of how much pester power a child exhibits. This suggests that parental engagement acts as a structural “circuit breaker,” not simply as a response to conflict. The findings also point to the need for marketers and regulators to look more closely at in-store promotional environments rather than advertising alone. Supporting parental engagement may do more to encourage healthy family shopping experiences than trying to manage children’s demands after they arise. The implications for FMCG marketers, public health regulators, and family consumer educators in emerging-market contexts are discussed.

## 1. Introduction

Parental power is no longer the only factor shaping how families decide to consume. As children’s access to digital media, peer networks (Clarke, 2009), and retail marketing increases, they play a much more significant role in determining what their family consumes—especially in relation to food and drinks (Mcleay & Oglethorpe, 2013; Marshall, 2005). Consequently, authority over how a family will consume has evolved into a more dynamic and negotiation-based model than traditional authority models.

India offers a unique opportunity to analyze these changes in the consumer space (Chaudhary, 2015). India provides a particularly instructive empirical theatre: a packaged food and beverage sector projected to exceed USD 70 billion by 2025, a retail landscape undergoing rapid organized-format expansion, and a generation of digitally native children who encounter food brands as fluidly online as in the supermarket aisle. Federation of Indian Chambers of Commerce and Industry (FICCI, 2022).

This specificity of the context is further highlighted by some recent studies in economies of South Asia adjacent to one another (Senevirathna et al., 2022); for instance, pester-power dynamics in urban Pakistani (Danish et al., 2023) families were observed to significantly enhance children’s influence on food purchases, in close cultural proximity to parental authority and within collectivist family structures, typically a pattern that holds for the familial context in India and calls for models grounded in the specific regions rather than the uncritical transplantation of findings from the West. In addition to rapid urbanization and rising disposable incomes, food and beverage markets continue to expand across the country. Children today are being raised in a world connected to various channels for brand messaging. They will encounter these messages through social media, online video games, and/or apps to learn more about products they want before they buy them, and will frequently see them when they go into grocery stores or other retail environments. As a result, children today have a much greater ability to influence their families’ purchases than in the past, and it is increasingly important to understand how this influence works.

Consumer Socialization Theory (Ward, 1974; Moschis & Churchill, 1978) (hereinafter referred to as CST) can be used to understand how kids have an effect on their parents’ purchasing decisions. Traditionally, CST has seen socialization as occurring only one way, from parent to child. The more recent research shows that children also influence their parents; for example, kids form brand loyalties and preferences through interactions with peers, media, and other experiences, and they bring those preferences into family purchasing decisions. Therefore, reverse socialization illustrates how children’s input is becoming increasingly important in shaping families’ buying behavior (Ekstrom, 2007; Watne & Brennan, 2011).

An obvious example of reversed socialization is when children repeatedly exert pester power on their parents to get them to buy products. Previous research has demonstrated that exposure to advertising, store promotional activities, and peer interaction leads to pester behavior, but the processes and mechanisms of how these influences are connected to pester behavior have not been adequately elucidated. In particular, there has been little research on the peer effect as a vehicle for establishing behavioral validity of promotional stimuli, thereby limiting understanding of how marketing stimuli lead to the development of a relationship with pester power.

Another limitation is that most research on the topic fails to highlight the positive aspects of pester power. Some of the research focuses on family conflict, parental strain, and shopping stress as they relate to family dynamics; however, these findings provide only a partial picture of family consumption dynamics. For example, parents do not always respond to children’s requests passively or defensively. Through active communication, guidance, or involvement in purchase opportunities, parents can transform potentially hostile shopping encounters into positive learning experiences; however, the positive aspects of parental engagement have received little attention in current theoretical models.

Another significant gap in the literature on pester power and consumer socialization pertains to context. The majority of empirical studies on these issues were conducted in Western countries; comparatively few have been conducted in an emerging market such as India. In India, there are also strong familial relationships, which exist alongside rapid modernization of retail, as well as increasing exposure to global marketing methods. It is therefore vital to understand how these two forces interact to create a more complete understanding of the development of contextually relevant theory surrounding family consumption patterns.

Much of the literature casts parental insistence in a negative light, associating it with conflict between parents and children, parental stress, and financial strain, especially in low-income households (O’Neill et al., 2014). A smaller body of research, however, suggests that insistence itself does not determine the outcome. What matters is how parents respond: the experience may become conflictual, or it may turn into something constructive that parents and children share (Watne & Brennan, 2011).

To address these gaps, this study develops and examines a dual-pathway model grounded in Consumer Socialization Theory. The model is used to explore in-store promotional influences on children’s ability to use their pestering power—both directly and indirectly through peer influence. Furthermore, it investigates whether strained parent–child interactions can act as an independent pathway to facilitate positive co-shopping experiences. Accordingly, the study addresses the following research questions:

**RQ1:** To what extent do in-store promotional stimuli amplify pester power, both directly and through peer influence as a mediating mechanism?
**RQ2:** What is the structural magnitude of pester power’s effect on parent–child conflict in food purchase contexts?
**RQ3:** Does proactive parental engagement constitute a structurally independent “circuit-breaker” pathway that generates positive co-shopping experiences independently of pester-power dynamics?

The remainder of this article is organized as follows. Section 2 develops the theoretical framework and hypotheses; Section 3 details the sample, measures, and analytical approach; Section 4 reports the measurement and structural model results; Section 5 discusses the findings, their theoretical contribution, and their limitations; and Section 6 concludes.

## 2. Theoretical Framework and Hypothesis Development

### 2.1. Consumer Socialization Theory and the Reverse Socialization Turn

The main concept of this research was shaped by the theory of consumer socialization. Ward’s (1974) original definition of socialization as learning how to function in the marketplace (attitudes, knowledge, and skills) was then changed by Moschis and Churchill (1978) to include three major sources of socialization (family members, peers, and mass media) that are used to create how consumers learn to shop. A review by John (1999), a comprehensive recap of the literature on this topic, demonstrated that children are active agents in cognitive development who interpret and negotiate cues from their environment rather than passively taking them. Finally, a recent study by Muhammad et al. (2019) on the significance of all three socialization agents, conducted in Pakistan-based settings, validates prior work.

The concept of bidirectional reverse socialization of children and parents was introduced by the reverse socialization theory (Ekstrom, 2007; Watne & Brennan, 2011; Jiao & Wei, 2020; Essiz & Mandrik, 2021). This view suggests that children who are socialized into brand preferences through peer networks (Clarke, 2009; Danish et al., 2023) and promotional environments then become agents of socialization by transmitting market awareness (Essiz & Mandrik, 2021), digital literacy, and product knowledge to their parents, thus exerting influence on purchasing decisions in families based on media-derived and peer-supported product knowledge (however, contrary to children’s “pestering power” image described in earlier literature as the basis of their strategic influence (Nicholls & Cullen, 2004; Buijzen & Valkenburg, 2008; Askelson et al., 2019), recent studies have applied this reverse socialization perspective to digital settings as well). Children encounter promotional content not just on television or in stores, but also through social-media influencers and platforms including YouTube, Instagram, and TikTok. These exposures have been shown to influence food and beverage preferences and requests among children and adolescents (Potvin Kent et al., 2024; Mc Carthy et al., 2022; Evans et al., 2023; Maksi et al., 2024; Lafontaine et al., 2025). This form of consumer socialization is related to, yet separate from, the in-store and broadcast-advertising stimuli examined in the present study. It is considered later as both a limitation and an avenue for future research.

This study adds to the body of research on conflicts between parents and children (CST) by developing an integrative model that traces the sequence of steps from environmental stimuli from the market, through the mechanisms where influential peers affect pester power, and finally resulting in one of two ways—representative of the two forms of parent/child conflict—either through conflict with the child or through positive shopping experience with the child (co-shopping). A critical aspect of this model is the engagement of parents with their child in order to “close the circuit” associated with co-shopping rather than engaging in either adversarial conflicts or negative shopping experiences with their child. While previous research published by one researcher (Ekstrom, 2007) suggested this fundamental aspect qualitatively, large empirical studies have not been conducted that support this construct.

### 2.2. Market Stimuli: The Primacy of In-Store Promotions

Researchers have identified three main types of market stimuli that can encourage children’s preferences for food products and influence them to request that their parents buy them: packaging and branding cues, advertising/media exposure, and in-store promotional environments (Binder & Matthes, 2023). Although the other two types of stimulus have been studied more extensively, so far, there has not been much structural investigation done to compare the effectiveness of in-store promotions as compared to the other two types of stimuli, despite evidence showing that in organized retail settings, they are quite powerful in encouraging children to ask for food products to be purchased (Wakefield & Inman, 2003; Wilson & Wood, 2004; Lam et al., 2018).

The majority of the academic literature on the ways in which companies market their food products to young children uses television as the primary means of advertising (Buijzen & Valkenburg, 2003; Calvert, 2008; Kelly et al., 2019; Gunardi et al., 2023; Verma & Kapoor, 2004). Television advertising often has its primary effects on children’s food purchase decisions through a variety of cognitive pathways (Nairn & Fine, 2008), including remembering a brand, associating a positive relationship with the product advertised, and framing the type of food that is aspirational or desirable (McNeal, 1992; Wakefield & Inman, 2003). Similarly, once children have tablished a level of brand familiarity, they can develop brand recognition before being able to read or write (McNeal, 1992). Children are influenced at the retail store positively, immediately, and in the presence of their parent(s) by how free items/sets of products are bundled together, how fun products are packaged (i.e., themed to a television show, game, etc.), how candy and sugary snacks are placed next to checkout/open and readily available for picking up, and how promotional signs are displayed at the point of purchase (Wakefield & Inman, 2003; Mulligan et al., 2023).

The theorists behind the current research predicted, based on this distinction, that advertising and packaging would increase product desirability through exposure mechanisms, whereas in-store promotions would have both direct and indirect impacts on pester power. In-store promotional displays will be an irresistible, at-hand stimulus and will give the promoted product social value via peer influence, making it a prominent possession among peers. Initial analyses of the data revealed that packaging did not have a significant direct path to pester power (β = 0.014, ns), nor did advertising (β = −0.022, ns). This is consistent with Christino et al. (2019), who found that packaging operates through preference formation rather than immediate solicitation. This supports the view that packaging and advertising primarily create an environment in which brand-relatedness occurs (through socialization) rather than directly prompting the solicitation of products. 

To maintain model parsimony, both non-significant direct paths were removed from the final structural model, with advertising having an indirect influence on pester power through peer influence. The following hypotheses are therefore advanced:

**H1a.**  *In-store promotions (C4) exert a positive direct effect on pester power (C6), over and above the indirect effect mediated by peer influence.*

**H1b.**  *In-store promotions (C4) positively influence peer influence (C5).*

**H1c.**  *Advertising and media exposure (C3) positively influence peer influence (C5).*

### 2.3. Peer Influence as the Social Amplification Mechanism

Harris’s (2009) study proposed a new way of thinking about children’s preferences and behavior as being shaped by their peers via socialization. Specifically, he argued that through peer socialization, children tend to prefer and behave in ways that they see their peers prefer or behave. In middle childhood, socialization via peers has a far greater cumulative impact on children’s preferences and behaviors than socialization via parents. In addition, peer influence works like a circuit around social validation because when children experience an advertisement for a marketed product (e.g., a brand of food) that they believe their peers already have, want or approve of, that advertisement has more legitimacy and urgency to them than the advertisement alone will have (Chaplin & John, 2007; Moschis, 1985; Zhou et al., 2023). In the case of a child seeing an in-store promotion for a branded food product, that child does not only want the product, but also the social status that comes with owning the product based on the peer reference group; therefore, the peer reference group has already established the value of the product and increased the likelihood that the child will purchase it.

Though the pathway of social amplification has been conceptually recognized in the CST literature (Moschis, 1985; John, 1999), this avenue of research has never been structurally examined in a comprehensive pester-power model based on a large sample. Notably, Gketsios et al. (2022) found no significant peer effect on junk food consumption in a Greek school-based sample, suggesting that peer influence on food choice may be context-activated and operative primarily in settings where peer norms are explicitly stimulated by promotional environments rather than universally present. 

The current model has considered the influence of peers as an endogenous factor, one determined by the combination of advertising and in-store promotional stimuli, which in turn contribute to the determination of pester power, thereby allowing this mediating process to be examined directly. The peer influence construct includes obtaining information about products and where/how to obtain them, and obtaining products to gain peer approval, confidence, and a sense of belonging. The following hypotheses have been created to explore this:

**H2a.**  *Peer influence (C5) positively influences pester power (C6).*

**H2b.**  *Peer influence (C5) mediates the effect of in-store promotions (C4) on pester power (C6), such that the indirect effect is positive and statistically significant.*

**H2c.**  *Peer influence (C5) mediates the effect of advertising (C3) on pester power (C6), such that the indirect effect is positive and statistically significant.*

### 2.4. Pester Power and Parent–Child Conflict

When parental resistance is present, pester power creates the conditions for intra-familial conflict. It has been established that the frequency of requests for household goods creates parent–child conflict as a proximate predictor in the food environment, mediating the distal effect of advertising-related materialism (Buijzen & Valkenburg, 2008). The different types of pestering strategies can include an inadvertent pattern of using repetitive requests to stay below the relative irritation threshold, bargaining, using expressions of affection with a contingent upon compliance element, and, in a minority of cases, using purposeful deception as a method to create conflict within the home when parents are engaged in gatekeeping regarding health-related purchases (Nicholls & Cullen, 2004; Kelly et al., 2019).

In the context of Indian families, conflicts have additional social implications. A parent’s level of embarrassment about their children’s pestering behavior in public is an extreme, highly culturally based interpersonal issue, as noted by Kaur and Singh (2006).

The Conflict Outcomes are defined as product ownership, aggressive behaviors, dissatisfaction, and public embarrassment, which serve as measures for an empirically based classification of the types of conflict that can occur between families in public and private spaces and in the retail food environment. The hypothesis is as follows:

**H3.**  *Pester power (C6) positively predicts parent–child conflict (C7).*

### 2.5. The Circuit-Breaker Pathway

Parental Engagement and Positive Co-Shopping. Although researchers have identified an adversarial dynamic of conflict between children and their parents, there is limited empirical evidence to support this conclusion. Instead, both the consumer socialization literature, as well as the reverse socialization literature, suggest that a certain subset of parents do not view child-driven consumption experiences as battles to be fought (won or lost), but instead, as opportunities for co-learning, collaborative decision-making, and furthering relational closeness (Ekstrom, 2007; Watne & Brennan, 2011). As such, the enabling factor for this new perspective of the parent–child relationship is parent engagement, as evidenced by three characteristics: curiosity about their child’s consumption world, willingness to participate in co-shopping occasions and a significant amount of quality time devoted to knowing their child’s hopes and/or peer-group dynamics.

This is referred to as the circuit-breaker route since it not only diminishes hostility but also interrupts it from within the adversarial viewpoint and substitutes co-participative for unilateral, authority-based relationships. The circuit-breaker analogy demonstrates a difference in structure vs. scale; engaged parents are not only more likely to tolerate being “pestered”, they are also functioning in a completely different type of relationship with daughters in terms of how they view the market knowledge of their children as an asset to be recognized rather than as a burden to be resisted. Therefore, whilst there is a structural prediction that children who are engaged with their parents will have more satisfaction during shopping trips together, it is independent of/orientable to the pester-power dynamics in the conflict route.

There are three components of parental engagement (C1): willingness to co-shop, quality investment in time spent together, and openness to understanding their kids’ interests. The positive co-shopping (C8) component consists of parental satisfaction, pride in their children’s ability to shop successfully, and the actual experiential quality of shopping with a child as a shared experience. The final two hypotheses are designed to complete the theoretical framework:

**H4a.**  *Parental engagement (C1) positively predicts positive co-shopping experiences (C8).*

**H4b.**  *The parental engagement → co-shopping pathway (H4a) operates independently of pester-power intensity, such that pester power (C6) does not predict positive co-shopping (C8) when parental engagement is controlled.*

## 3. Methodology

### 3.1. Research Design, Sample, and Data Collection

The research employed a quantitative, cross-sectional survey design to survey urban Indian parents of at least one child aged five to twelve years living in Tier I (e.g., Kochi, Bengaluru, Chennai) and Tier II metropolitan areas. This population was selected on theoretical grounds: organized retail penetration and dual-income households are at their highest in these urban centers, creating the exact promotional environment and time-challenged parenting situations that theory predicts will create the greatest pester-power dynamics (Kaur & Singh, 2006; McNeal, 1992). Convenience and snowball sampling produced 183 responses; 14 were eliminated based on a screening filter (respondents without qualifying children), yielding an analytically viable sample of = 169. Data were collected entirely online via a structured Google Forms questionnaire, distributed through school parent networks rather than through in-store or telephone administration.

The adequacy of the sample for PLS-SEM is determined through Cohen’s (1992) power analyses, whereby the structure of the construct receiving the greatest number of paths—pester power (C6)—requires a minimum sample size of 85 in order to show a significant (R^2^ > 0.10, α = 0.05, power = 0.80) result. Since our final sample was 169, we have a sufficient power to detect an effect for all of our proposed paths, as well as for trial-mediated paths via bootstrap analysis.

### 3.2. Measures and Instrument Development

The survey included a total of 82 items, which consisted of an eligibility screen, 11 demographic questions, and 70 five-point Likert questions (Strongly Disagree = 1 to Strongly Agree = 5) representing each of the eight theory areas. There was also a multi-answer question relating to food products that were requested the most by respondents’ children. The Likert questions were based on previous studies regarding consumer socialization and “pester power” (Buijzen & Valkenburg, 2003; Nicholls & Cullen, 2004; Moschis & Churchill, 1978) but had been modified for the Indian FMCG retail market. As an example, many of the Western brands were replaced with brand names of Indian packaged food items that are known in India. Of the 70 items, 22 came directly from existing validated scales, with only the brand or context changed. Another 31 were reworded for cultural and linguistic clarity, including simpler phrasing for respondents who were not native English speakers. The remaining 17 were written specifically to capture retail phenomena found in India, such as point-of-sale bundling common in Indian organized retail, that the source scale did not cover.

To validate the content, we received input from five university-based marketing research scholars and two consumer psychologists (experienced with child consumers) before conducting a cognitive pilot study (n = 30) to confirm participants’ understanding of the items and gather preliminary measures of reliability using Cronbach’s alpha (ranging from 0.63 to 0.89).

### 3.3. Item Purification and Measurement Model Quality

After obtaining data, a systematic protocol was used to purify items from the measurement model to meet Fornell and Larcker’s (1981) and Hair et al.’s (2019) recommended threshold for assessing convergent validity, which is that the average variance extracted (AVE) should be equal to or greater than 0.50. The item purification process involved an iterative method—for each of the eight constructs, the outer loading value associated with each item was evaluated. The item with the lowest outer loading value was deleted, the path model using partial least squares (PLS) was recomputed, and the AVE was recalculated. This process continued until all eight of the constructs contained a minimum of 36 items (70 items originally).

We treat the purification process, including the removal of the two non-significant direct paths reported below, as an exploratory refinement stage, distinct from the confirmatory testing of the formally hypothesized model. It preceded and helped shape the confirmatory bootstrap estimation of that structural model, presented in this paper. Both non-significant paths are retained in Table 4 rather than omitted without explanation.

The following two path-significance results are reported here only to explain the item- and path-purification decisions that shaped the final measurement model; the confirmatory structural-model results these decisions fed into are presented in full in Section 4.2.

Two paths—packaging pester power and ads to pester power—were initially non-significant (β = 0.019 and −0.009, respectively) and therefore removed prior to finalizing the structural model. Removal of non-significant direct paths was done based on theoretical reasoning: they act further upstream through peer validation and brand socialization rather than as immediate triggers for pester behavior. This theory is further supported by the significance of the advertising indirect path (through peer influence: indirect β = 0.066, *p* = 0.008). Non-significant direct paths were also removed because they did not fit into the theoretical distinctions between environment-based market stimuli and exposure-based market stimuli.

Table 1 presents measurement quality metrics for the final eight-construct, 36-item model. Each construct meets the three psychometrics criteria outlined by Hair et al. (2019) for reflective PLS-SEM: Table 1 outer loadings for all constructs are >0.60 with a range of 0.563 to 0.886 (grand mean = 0.760), resulting in meeting indicator reliability; Table 2 composite reliability (CR) among the eight constructs ranges from 0.795 to 0.914 (all >0.70), thus meeting internal consistency reliability; and Table 3 average variance extracted (AVE) ranges from 0.536 to 0.643, thus all constructs meet convergent validity with AVE values > 0.50. Discriminant validity is established based on the heterotrait–monotrait (HTMT) ratio proposed by Henseler et al. (2015); the highest HTMT value was found to be 0.806 (between pester power and conflict outcomes), which is below the conservative threshold (0.85), demonstrating that each construct represents a different, theoretically relevant dimension of the pester-power ecology.

### 3.4. Analytical Method: Justification for PLS-SEM

The selection of Partial Least Squares Structural Equation Modelling (PLS-SEM) as the principal analytical mode was predicated on four principles (Zeng et al., 2021), each being an alignment between the analytical assumptions and characteristics of the data and objectives of the study.

First up, sample size of the current study: 169 cases do not meet the necessary threshold of 200+ cases to ensure stable maximum likelihood estimation (MLE) using Covariance-Based Structural Equation Modelling (CB-SEM) (Hair et al., 2019). The variance-based estimation of Partial Least Squares Structural Equation Modelling (PLS-SEM) is independent from distribution and can produce reliable results using a sample size of 30–100 for each endogenous construct—making it an appropriate technique for the design of the present study.

Second, research objective orientation. PLS-SEM optimizes the explained variance of endogenous constructs—this is the exact research objective of this study: to quantify the structural effects of market stimuli, peer pressure, pester power, and parental engagement on consumption at the household level. CB-SEM optimizes the reproduction of the observed (covariance matrix) and is more appropriate for theory-confirming studies where global model fit is the primary measure of success.

Thirdly, distributional assumptions: Likert-scale cross-sectional field survey data in developing countries often do not follow a normal distribution, violating CB-SEM’s assumption of multivariate normality. Non-parametric bootstrapping using PLS-SEM does not require multivariate normality, which means it produces stable/robust inferential statistics for models regardless of the data’s distributional form (Ringle et al., 2015).

Fourthly, reflective measurement. All eight constructs are reflective (Mode A) and are treated as interchangeable indicators of the underlying latent construct. This specification is grounded in theory; pestering behaviors, expressions of peer influence, and items representing satisfaction from co-shopping all represent exchangeable realizations of the relevant latent dispositions, and this consensus has been confirmed by the factor-analytic structure determined in the pilot stage. 

All analyses were conducted using a custom NIPALS-based PLS-SEM implementation in version 4.x, following the estimation procedure detailed in Section 3.5 of this work, with statistical significance evaluated at α = 0.05 (two-tailed) throughout, except where bootstrap-based confidence intervals are reported directly.

### 3.5. Estimation Procedure and Bootstrap Inference

The PLS used NIPALS-type iterations to update outer weights via centroid averaging and to estimate the Mode A (reflective) outer weights. Convergence was reached at a tolerance level of 1 × 10^−8^ and usually took between 50 and 80 iterations. Each iteration of outer weight standardization applied unit variance, thereby producing latent variable scores that are scale-invariant for subsequent understanding of the latent constructs’ paths. The estimation of path coefficients was accomplished using ordinary least squares regression, in which each intrinsic construct score was regressed on its theoretically proposed predictors within the model structure.

In this manuscript, the authors used a non-parametric bootstrap to estimate the complete structural model at B = 5000. The coefficients, effect sizes (f^2^), and R^2^ values reported in Table 4, Figure 1, and Table 1 are estimated from an independent reconstruction of the raw response data. This assigns each of the 70 survey items to its construct using the section structure of the original data-collection instrument—a dedicated parental engagement/family environment section, a combined packaging/brand/advertising/promotion section, and a combined peer influence/store environment/pester power/conflict/co-shopping section together with the construct definitions in Section 2.1, Section 2.2, Section 2.3, Section 2.4 and Section 2.5 to split the two combined sections into their constituent constructs. All confidence intervals reported are bias-corrected, with 95% confidence.

### 3.6. Common Method Variance Controls

Since predictor and criterion variables were collected from a single respondent at the same time, common method variance (CMV) may be a confounding issue (Podsakoff et al., 2003). The use of certain procedural controls (e.g., separating antecedent and outcome constructs by using multiple sections on the questionnaire) and the inclusion of reverse-coded buffer items at least partially alleviate this concern. The Harman single-factor test (Harman, 1967) indicated 31.4% of the total variance could be explained by a single factor, therefore below the CMV concern level (i.e., 50%). Furthermore, the marker-variable method (Lindell & Whitney, 2001) was used to evaluate whether adjustments for CMV would affect the hypothesized statistical significance or pattern of relationships. Overall, these controls suggest that CMV is unlikely to be a systematic confounding variable in this dataset.

## 4. Results

### 4.1. Measurement Model

As shown in Table 1, all eight constructs satisfy the reflective-model thresholds recommended by Hair et al. (2019) and Fornell and Larcker (1981). For each construct, outer loadings, composite reliability, and AVE fall within acceptable ranges. Discriminant validity is also supported, both by the HTMT ratio (Table 1 note) and by the Fornell–Larcker criterion (Table 2). In-Store promotions (C4, AVE = 0.506) comes closest to its threshold, which is consistent with the narrower item pool retained for that construct after purification (see Section 5.6). The remaining constructs are comfortably above threshold, supporting the use of this measurement model in the structural analyses explained in forthcoming sections.

### 4.2. Structural Model: The Pester-Power Conflict Pathway

Table 4 reports all path coefficients, bootstrap standard errors, and t-statistics for the reduced structural model, showing that the pester-power-to-conflict pathway holds with a high degree of confidence.

In-store promotions have the largest impact on peer influence (the direct influence) and show the greatest peer influence on pester power (both statistically significant) (H1a; H1b). The advertising also shows a statistically significant effect on peer or pester influence (H1c). Additionally, peer influence shows a statistically significant effect in predicting the pester or peer influence (H2a). Overall, the in-store promotions and advertising together explain 33.5% of the variance of peer influence. Store promotions, advertising, packaging, and peer influence are collectively responsible for 21.6% of the variance in pester power, R^2^ = 0.216. Pester power was also found to be the significant and exclusive predictor of parent–child conflict, β = 0.608, SE = 0.053, t = 11.375, *p* < 0.001, CI [0.500, 0.707], f^2^ = 0.585 (large), R^2^ = 0.369, thus confirming H3 with a large effect size. The relatively modest R^2^ for pester power (0.216) suggests that the market-stimulus and peer-influence predictors included here leave much of its variance unexplained. Other factors may be involved, including children’s individual temperament and willingness to negotiate, household spending norms, and exposure to digital/social-media channels that this survey did not capture (see Section 5.6).

### 4.3. Mediation Analysis: The Social Amplification Mechanism

According to Hayes (2018), both market-stimulus-mediated effects of peer influence on pester power are statistically significant, with 95% confidence intervals (CIs) that do not include zero; thus, both meet the criteria for mediational inference. In addition to supporting H2b, the in-store promotional effect on pester power through peer influence is statistically significant with the indirect β = 0.105, SE = 0.039, t = 2.721, *p* = 0.007, and 95% CI of [0.033, 0.185]. The advertising effect on pester power through peer influence is also statistically significant, having an indirect β = 0.066, SE = 0.032, t = 2.103, *p* = 0.008, and 95% CI of [0.014, 0.136], thereby supporting H2c. Furthermore, because there is no significant direct path from advertising to pester power, advertising’s effect on pester power is fully mediated by peer influence.

### 4.4. The Circuit-Breaker Pathway: Parental Engagement and Co-Shopping

H4a was supported because parental engagement (C1) has a positive and statistically significant effect on positive co-shopping experiences (β = 0.527, *p* < 0.001, f^2^ = 0.341 [medium-to-large], R^2^ = 0.284). H4b was supported because pester power (C6) does not have a statistically significant effect on positive co-shopping shopping (β = −0.016, *p* = 0.750, f^2^ = 0.000). The circuit-breaker category is achieved based on the evidence: parental engagement produces co-shopping satisfaction through a completely different pathway than the pester-power dynamics affecting the co-shopping conflict relationship. Engaged parents are not only less likely to have a co-shopping conflict, but they are also in a relational framework that is different than parents that are pestered. As a result, positive co-shopping outcomes will occur regardless of the amount of pester power in the home. This non-significant path suggests that no statistical association between pester power and co-shopping was detected in this sample. On its own, though, it does not establish that the two constructs are theoretically independent. That stronger claim would need support beyond a single non-significant cross-sectional path coefficient, such as experimental manipulation or replication in other samples.

## 5. Discussion

### 5.1. In-Store Promotions as the Primary Socialization Agent

In the literature of food marketing to children, pester power is most influenced by in-store promotions as compared to advertising and packaging, which reflects a shift away from the traditional focus in both legislative and academic studies of food marketing to children on broadcast television advertising (Buijzen & Valkenburg, 2003; Kelly et al., 2019). In urban India, the physical store environment has the greatest impact as an immediate socializing agent, converting latent product desire (as primed by advertisements and peer norms) into requests to purchase on an ongoing basis through the presence of free gift baskets containing game characters, as well as the positioning of game-related merchandise on display at eye level and through other means that trigger memory recall when viewed in the store. The effect size of in-store promotions for peer influence (f^2^ = 0.198) indicates that the retail environment is not just the setting for direct pestering, but also that this shopping environment changes the peer-based standard of consumption against which peers compare their consumption, thereby creating a multiplier effect of social reinforcement that continues beyond the immediate shopping experience.

This study leads regulators to shift their policy focus from social responsibility to the regulation of the advertising of products that children might want; advertising is only one of many inputs into the pester power/conflict chain. This is in line with the work of Driessen et al. (2022), which directly establishes that parents underestimate the level and impact of food marketing, especially in digital ecosystems, which supports why policy intervention at the point of sale is needed. Similarly, Ertz and Le Bouhart (2021) provide a macro-marketing theoretical framework for why junk food marketing constitutes a systemic public health problem requiring structural intervention.

Beyond controlling advertising content, this also implies a distinct policy lever: point-of-sale promotional codes for retailers, analogous to existing broadcast-advertising regulations, targeting the in-store promotional environment identified here as the dominant driver of pester power.

### 5.2. Peer Influence: From Contextual Factor to Structural Mediator

Peer influence has been established as an important structural mediator of pester power, modifying the theoretical view of the relationship between market stimuli and pester-power behavior. Previous models have treated peer influence as either a moderator or an exogenous context; the current study finds that peer influence is best viewed as part of a loop-signal circuit for amplification via convergence from advertising and in-store promotions. Peer influence serves as both an input and output device used by consumers when soliciting purchases from the social group; pester-power behavior has been determined jointly by exposure to advertising and in-store promotions as well as the peer group; therefore, pester-power behavior is not initiated by market stimuli in isolation; instead, pester-power behavior is facilitated through the filtering of market stimuli by peer influence, creating urgency in purchase solicitation. Full mediation of the advertising effect on pester-power behavior by peer influence (i.e., a non-significant direct effect with a significant indirect effect) has critical implications for theoretical understanding: the results indicate that the social validation component of broadcast advertising constitutes the sole path to pester power through the process of social validation rather than the individual cognitive priming of consumers. Therefore, the findings of the current study provide important insights for how researchers model the impact of media on family consumption patterns.

This model mirrors how quickly India’s retail sector has become organized. As modern sales formats spread and disposable incomes rise, the prominence of in-store promotions may signal a product’s value within a social group. A store, then, is not merely somewhere to buy things. Its setting makes consumer trends and desires visible, strengthening peer influence in the kind of mature, ambitious consumer economy described by FICCI (2022).

Placed alongside other emerging-market and cross-cultural research on children’s purchase influence—including Indian samples outside the FMCG-food domain (Ansari et al., 2023; Nain, 2021; Işın & Alkibay, 2011) and an Arab-region comparison of parents’ and children’s perceptions of influence (Ghouse et al., 2020)—the study suggests that pester power is driven by peer-mediated market stimuli, not advertising exposure alone. This broadly supports the view that, in collectivist, family-centered cultures, children exert influence through social and relational channels rather than through purely individual persuasion. It also extends the existing literature: to our knowledge, this is the first study to test peer influence structurally as a mediator, rather than as an independent correlate, of market stimuli in an Indian FMCG context.

### 5.3. The Anatomy of Conflict: Pester Power’s Large Effect

Pester power has a significant impact on driving family conflict (β = 0.608, f^2^ = 0.585, R^2^ = 0.369), as represented by its largest structural coefficient in family purchase solicitation (the basis for conflict in the food retail experience). This substantial coefficient of f^2^ = 0.585 indicates that pester power alone accounts for most of the variance in conflict outcomes, according to Cohen’s (1988) criteria; therefore, this is an important finding for both researchers and practitioners. This suggests that efforts to reduce family conflict in retail settings should focus on the antecedents of pester power, such as the promotional environment in the store, rather than addressing how parents resolve conflicts after requests have been made by children during the shopping experience.

Indian family life’s collectivist structure can intensify this effect. Asking publicly for a child is not seen merely as an individual want; with other people watching and judging in the store, it may also look like a claim on the family’s shared resources and status. A refusal, then, can feel like more than a financial choice. It may be understood as a breach of a relationship-based obligation. That helps explain why persuasion can turn into conflict—and, more specifically, public humiliation—more readily than it does in more individualistic consumer settings (Kaur & Singh, 2006).

### 5.4. The Circuit Breaker: Theoretical and Practical Significance

The pathway between parent involvement and co-shopping was the most significant theoretically and practically. Because the C6–C8 path was not significantly different from zero (β = −0.016, f^2^ = 0.000), we found evidence that co-shopping satisfaction is not the same as conflict, but rather it stems from a different kind of parenting behavior. Engaged parents (who spend time with their children to learn about how they consume things, are willing to co-shop, and are genuinely interested in what their children are trying to accomplish) do not just have less conflict with their children, but have a different way of relating to their children that is not influenced by the pester-power dynamic.

According to these results, the reverse socialization thesis (Ekstrom, 2007; Watne & Brennan, 2011) receives empirical support; co-learning is used by parents when they interact with children for their knowledge of the market, thus changing the shopping environment from a competition (for preference) between parents and children to one where both parties participate together, and parents can regard their children as sources of relational capital (rather than negative pressure) for the marketplace. This view reflects that children who are socialized into brand preferences through peer networks slowly transform into agents of socialization by transmitting market awareness; see Essiz and Mandrik (2021). The use of the term circuit breaker is theoretically accurate since engaging with children does not diminish the incidence of pestering by children (i.e., C1 → C6 does not exist), but rather it interrupts the pathway that leads from pestering to conflict at the family level, therefore altering the relationship dynamics from adversarial to collaborative.

This points to a practical intervention target: parent-engagement programs, such as structured co-shopping initiatives or brief communication-skills training offered through retailers, schools, or public-health outreach. The aim would be to strengthen parents’ willingness and ability to shop with their children. These interventions should improve the quality of the co-shopping experience directly, even if they do not reduce the level of pester-power activity in the household. Given the functions of these two independent pathways, creating engaged parents and mitigating predatory selling practices can occur in parallel at different points along this pathway.

This positive account of parental engagement has to be read alongside the harm associated with pester; it cannot establish the direction or temporal order of the observed associations. Consistent with earlier research on pester power in lower-income households (O’Neill et al., 2014), the present data indicate that it strongly predicts parent–child conflict, including aggressive behavior, dissatisfaction, and public embarrassment (H3). These are real costs to family wellbeing rather than mere background conditions for the circuit-breaker pathway. Children’s negotiation and influence tactics differ across contexts and are generally treated negatively in the literature (Ellis & Maikoo, 2018; Vel et al., 2016). The study’s mostly positive depiction of engaged co-shopping is not intended to overturn that position. Its focus is limited to in-store promotions, advertising, and packaging as market stimuli. Social media, influencer marketing, and other digital channels increasingly influence what children ask for. They fall outside the present model, though, and should be taken up in future extensions of the framework.

### 5.5. Theoretical Contributions

These findings extend Consumer Socialization Theory in three ways, though they are best understood as integrating and empirically validating mechanisms that had previously been conceptualized or only partly examined rather than as a wholesale refinement of the theory. The study first treats peer influence as an endogenous mediating mechanism through which promotional stimuli gain behavioral force. In doing so, it gives structural form to a pathway previously discussed only in conceptual terms (Moschis, 1985; John, 1999). It also offers evidence from an emerging-market setting that parental engagement leads to positive co-shopping experiences through a pathway structurally independent of pester-power dynamics. That finding supports the reverse-socialization literature’s argument that consumption exchanges between parents and children need not be adversarial (Ekstrom, 2007; Watne & Brennan, 2011). Third, the study tests a structural model using data from urban Indian families, providing empirical validation outside the Western settings where these constructs were largely developed and tested. Taken together, these contributions aim to assist marketers, policymakers, and consumer educators working to support healthier family consumption environments.

### 5.6. Managerial and Policy Implications

These findings call for different responses from different groups. For fast-moving consumer goods (FMCG) companies, the results suggest that redesigning in-store promotions, not increasing advertising investment, is the more effective way to affect both revenue and reputational risk. That conclusion supports an internal review of point-of-sale placement practices for products aimed at children. Public health regulators may have grounds to extend existing restrictions on audiovisual advertising (Dens et al., 2007) to point-of-sale promotional codes as well: in this sample, in-store stimuli had the strongest direct effect on parental pressure, while advertising did not. Family educators and consumer advocacy programs face a different implication. Because the parental involvement pathway is structurally independent (H4a/H4b), efforts to build co-buying skills do not need to wait for, or rely on, reduced exposure to parental pressure. Involvement can be encouraged separately, as a viable practice regardless of the family’s promotional environment.

### 5.7. Limitations and Future Research Directions

This study has several limitations. Because the design is cross-sectional, it cannot establish causal relationships. Longitudinal research is needed to determine the temporal direction between the pester power and conflict variables. The 169 participants (n = 169) were recruited through convenience and snowball sampling, and the sample was weighted toward urban, higher-income households in Tier I and Tier II Indian metropolitan areas. Rural and lower-income Indian households were not intended to be represented, so the reported effects should not be generalized beyond this population without further testing. Although the sample meets Cohen’s (1992) power-analysis threshold for the most heavily predicted construct in the model, n = 169 is still modest for an eight-construct structural model. Parameter estimates may therefore be less stable than desired, making replication with larger samples advisable. Fourth, item purification reduced the instrument from 70 items to 36 to meet the convergent validity thresholds. Although this improved the psychometric performance of each construct, removing roughly half of the original item pool may have narrowed their content coverage, or content validity. The issue is particularly apparent for in-store promotions (C4). Retaining one facet while discarding another—for example, tangible point-of-sale incentives but not general retail visibility or placement cues—could mean that the resulting measure reflects a different mechanism. Researchers seeking a broader operationalization of these constructs may want to revisit the items that were removed. Appendix A reports the complete item-level loadings and the sequence in which items were eliminated.

The two direct paths that failed to reach statistical significance—packaging → pester power and advertising → pester power—were removed from the final structural model following an initial exploratory run. Still, trimming a model in this manner can capitalize on chance and may not generalize consistently across samples (MacCallum et al., 1992). For that reason, both paths remain in Table 4, where they are reported as non-significant, rather than being omitted. Their removal should be understood as an exploratory, sample-specific decision, not as a confirmed theoretical conclusion. Fifth, the study did not gather the child’s direct opinions. Collecting data separately from parent–child dyads could provide further insight into how parent–child power dynamics shape pester power. Sixth, unless digital advertising stimuli social media, children’s influencer marketing, and app-based incentive programs are defined operationally and distinctly, researchers cannot determine how each type affects children’s pester power. Because children spent more time with digital media during the pandemic, future studies need to measure these channels accurately rather than combine them as a single independent variable.

## 6. Conclusions

This research identifies two separate pathways behind pester power: one driven by market and peer conflict, and another in which parental engagement acts as a circuit breaker. Pester power is thus not treated as a single, uniform phenomenon. In this urban Indian FMCG sample, in-store promotional activity is the strongest immediate market trigger, with peer influence substantially intensifying the effect of promotional stimuli. Parental engagement tells a different story: it predicts positive co-shopping experiences independently of pester-power intensity. Its role is structurally distinct, functioning as a circuit breaker rather than simply indicating that conflict is absent. These findings offer an initial, context-bound account, not a definitive or generalizable model of pester power. Longitudinal research should test the framework further, draw on more diverse socioeconomic samples, gather dyadic parent–child data, and examine digital/social-media marketing stimuli explicitly alongside in-store and broadcast chains. By establishing these findings, we change the nature of how we measure pester power from documenting an annoyance to creating process maps that show where interventions can be made in order to influence positive outcomes.

## Figures and Tables

**Figure 1:**
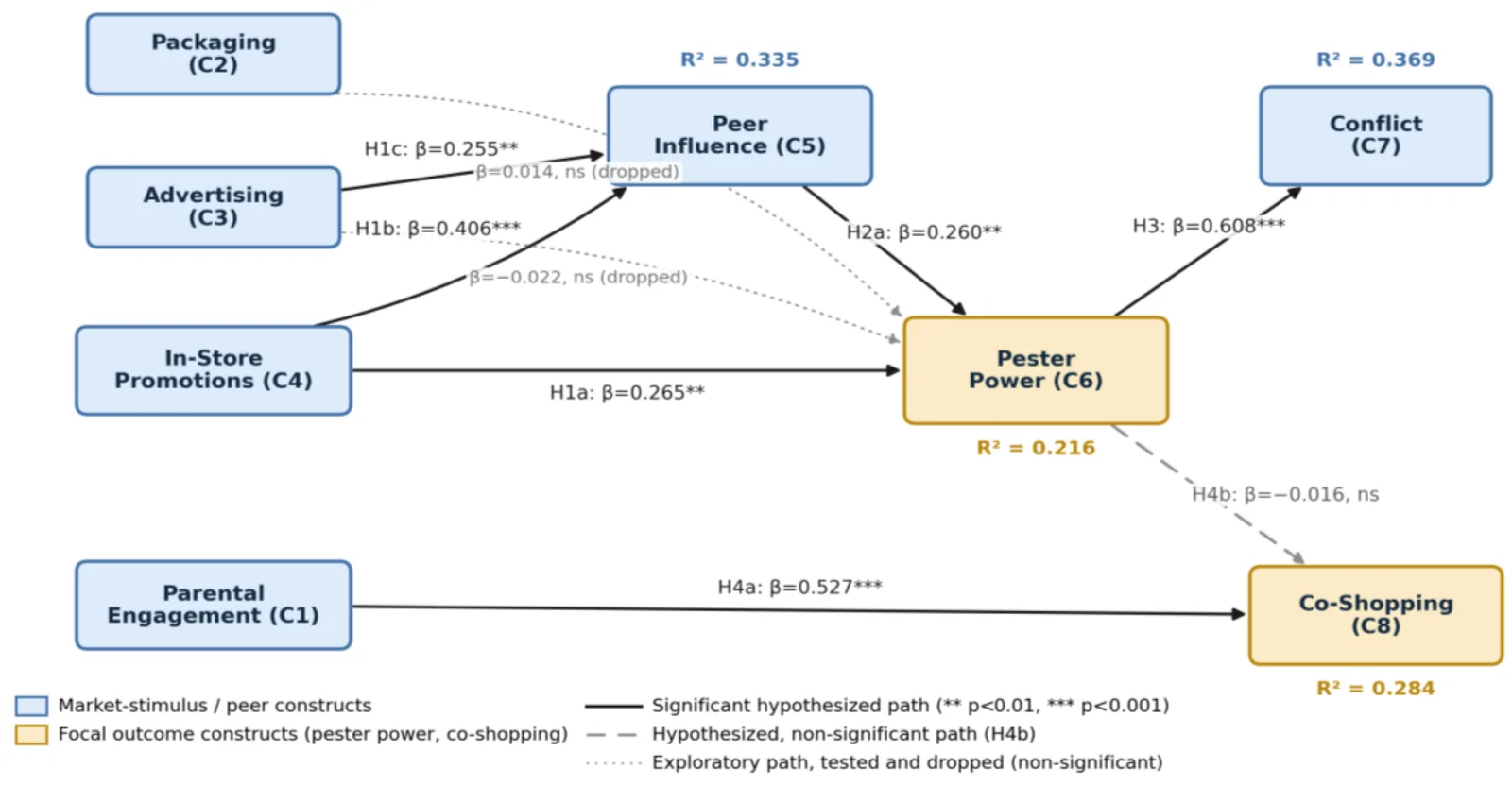
Structural model with standardized path coefficients (bootstrap B = 5000). Solid black arrows: significant hypothesized paths; grey dashed arrow: hypothesized but non-significant (H4b); grey dotted arrows: exploratory paths tested and dropped from the confirmatory model.

**Table 1:** Measurement model summary: final 36-item, eight-construct model. Item wordings for the final 36 indicators, and the 34 items removed during purification, are listed with their loadings in Appendix A.

Construct	Full Name	Items (Final)	α	CR	AVE	Loadings (Range)
C1	Parental Engagement	3	0.650	0.796	0.565	0.726–0.797
C2	Packaging & Brand	3	0.619	0.795	0.563	0.738–0.766
C3	Advertising & Media	5	0.823	0.876	0.585	0.731–0.798
C4	In-Store Promotions	4	0.708	0.821	0.536	0.684–0.827
C5	Peer Influence	6	0.875	0.906	0.618	0.686–0.835
C6	Pester Power	4	0.728	0.830	0.549	0.714–0.759
C7	Conflict Outcomes	5	0.789	0.857	0.551	0.563–0.832
C8	Positive Co-Shopping	6	0.886	0.914	0.643	0.671–0.886

Note. α = Cronbach’s Alpha; CR = Composite Reliability; AVE = Average Variance Extracted. All thresholds follow Hair et al. (2019): α > 0.60, CR > 0.70, AVE > 0.50. HTMT max = 0.806 (C6–C7) < 0.85 threshold (Henseler et al., 2015). All loadings reported are standardized outer loadings.

**Table 2:** Fornell–Larcker discriminant validity matrix. Diagonal values (bold) are the square root of AVE for each construct; off-diagonal values are inter-construct correlations. Every diagonal value exceeds every off-diagonal correlation in its row and column, satisfying the Fornell–Larcker criterion referenced above.

Construct	C1	C2	C3	C4	C5	C6	C7	C8
**C1**	0.817							
**C2**	0.038	0.750						
**C3**	−0.001	0.546	0.765					
**C4**	−0.083	0.474	0.510	0.732				
**C5**	0.039	0.439	0.462	0.536	0.786			
**C6**	−0.234	0.253	0.255	0.408	0.405	0.742		
**C7**	−0.281	0.212	0.286	0.379	0.407	0.608	0.742	
**C8**	0.362	0.188	0.073	0.058	0.137	−0.198	−0.118	0.801

**Table 3:** Full HTMT (heterotrait–monotrait ratio) matrix.

	C1	C2	C3	C4	C5	C6	C7
C2	0.161						
C3	0.091	0.733					
C4	0.144	0.703	0.665				
C5	0.101	0.586	0.531	0.670			
C6	0.499	0.362	0.319	0.564	0.492		
C7	0.444	0.305	0.362	0.516	0.483	0.806	
C8	0.648	0.281	0.152	0.150	0.161	0.266	0.184

Note: All 28 construct-pair values are below the 0.85 threshold (Henseler et al., 2015); the maximum C6–C7 = 0.806.

**Table 4:** Parsimonious structural model: path coefficients (Bootstrap n = 5000).

Hypothesis	Path	B	SE	t	*p*	95% CI	f^2^	Sig.
H1c ✓	C3 → C5 (Adv → Peer)	0.255	0.071	3.594	0.0002	[0.117, 0.396]	0.072	**
H1b ✓	C4 → C5 (Prom → Peer)	0.406	0.079	5.162	0.0002	[0.252, 0.562]	0.184	***
H1a ✓	C4 → C6 (Prom → Pester)	0.265	0.095	2.792	0.0088	[0.085, 0.446]	0.054	**
H2a ✓	C5 → C6 (Peer → Pester)	0.260	0.092	2.821	0.0076	[0.065, 0.432]	0.056	**
H3 ✓	C6 → C7 (Pester → Conflict)	0.608	0.053	11.375	0.0002	[0.500, 0.707]	0.585	***
H4a ✓	C1 → C8 (Engage → Co-Shop)	0.527	0.074	7.096	0.0002	[0.390, 0.681]	0.341	***
H4b ✓	C6 → C8 (Pester → Co-Shop)	−0.016	0.067	−0.247	0.750	[−0.148, 0.112]	0.000	ns

Note. β = standardized path coefficient; SE = bootstrap standard error; f^2^ = Cohen’s effect size; CI = bias-corrected 95% bootstrap confidence interval. *** *p* < 0.001; ** *p* < 0.01; ns = non-significant. Non-significant direct paths from C2 (packaging) and C3 (advertising) to C6 (pester power) were tested and excluded from the final structural model on theoretical grounds (Section 3.3); they are reported here rather than omitted. All path coefficients above, including these two, were estimated in a single, uniform re-estimation of the complete model at B = 5000.

## Data Availability

The data presented in this study are available on request from the corresponding author.

## Appendix A

**Table A1:** Complete 70-item measurement pool by construct, purification status, and outer loading. Of the original 70-item survey instrument, 69 were five-point Likert items assigned to one of the eight model constructs (the 70th item was a multi-select product-category question, not a Likert indicator, and is not part of this measurement model). “Loading” is the item’s outer loading in the retained 36-item model (Table 1) for retained items, or its initial loading in the full candidate pool before purification for removed items. All loadings shown are standardized outer loadings.

Construct	Item Wording (as Administered)	Status	Loading
C1	I spend quality time with my children to know about their interest and aspirations.	Retained	0.731
C1	My family spends time engaging in fun-filled activities together.	Removed	0.538
C1	I try to understand the activities that my child engages in in his/her free time.	Retained	0.726
C1	I am confident that my child will do well in the future.	Removed	0.565
C1	I feel good about his/her activities. (Higher you rate, higher is the degree of agreement).	Removed	0.448
C1	I tend to complain when I do not like something they bought for themselves.	Removed	0.116
C1	I used to tell them that they should not ask questions about things that kids like them do not normally buy.	Removed	0.083
C1	I tell them what things they should or should not buy.	Removed	−0.067
C1	I talk to my child about why I buy the brands or products I purchase.	Removed	0.531
C1	I am happy to take my kid shopping with me.	Retained	0.797
C1	I often avoid taking my kids shopping with me.	Removed	0.507
C1	My child and I talk about buying things we see or hear advertised.	Removed	0.561
C1	I encourage my child to make requests for products for their own use.	Removed	0.550
C2	My child chooses products that have used interesting and attractive colors in their packaging and persuades me to purchase.	Retained	0.738
C2	My child gets attracted to products with a package that makes good use of cartoon characters (e.g., Winnie the Pooh and Mickey Mouse) and persuades me to purchase.	Removed	0.532
C2	My kids get attracted to products that have a familiar brand name to them and persuade me to purchase.	Retained	0.766
C2	When I take my child shopping, a food product with a familiar brand name is one of his/her first choices and I am persuaded to purchase.	Retained	0.747
C2	My child persuades me to buy a product that is frequently used at home.	Removed	0.539
C2	I find child-oriented packaging to be a nuisance when shopping with my kids.	Removed	−0.589
C2	I find child-oriented packaging to be helpful when making purchase decisions in supermarkets.	Removed	−0.120
C3	Products that are advertised in vibrant colors e.g., red, blue, green, orange, and yellow, influence my child to request of me to buy that product.	Removed	0.638
C3	Functional attributes of products, e.g., popcorn and Maggi in two minutes, in the advertisements influence my kid to make a purchase request.	Removed	0.577
C3	Funny advertisements influence my kids to make purchase requests.	Retained	0.731
C3	Demonstration of the product and its benefits in the advertisements makes my child make a purchase request.	Removed	0.534
C3	Jingles explaining product characteristics and benefits help my child to recall the product as well as persuade me to purchase.	Retained	0.741
C3	Rhythms of jingles help in my kid recalling the product as well as in persuading me to purchase it.	Retained	0.783
C3	The frequency of advertisements on television makes my kid persuade me to buy the product as they give information about the product.	Retained	0.798
C3	The frequency of advertisements on television makes my kids persuade me to buy the product as they help them to recall the product.	Retained	0.768
C3	I have observed my kids getting connected to the child in the advertisement.	Removed	0.578
C4	My kids persuade me to purchase products that have puzzles on product packaging.	Removed	0.498
C4	My kids persuade me to purchase products that have games on product packaging.	Retained	0.720
C4	My kids persuade me to purchase a product that comes packaged with free gift/s.	Retained	0.827
C4	My kids persuade me to purchase products that have gift vouchers.	Removed	0.561
C4	My kids prefer buying products that are small in size, e.g., small pack of biscuits, chocos that are in the shape of savories, stars, etcetera.	Retained	0.684
C4	Products in retail stores help my kids in persuading me to buy them as they are in their reach, e.g., biscuits.	Removed	0.584
C4	Products in retail stores help my kids in persuading me to buy as they are displayed in such a place that they can see and select.	Removed	0.572
C4	Products in retail stores help my kids to persuade me to buy as they help them with recalling a product.	Removed	0.615
C4	Products in retail stores help my kids in persuading me to buy as they are displayed on the billing counter, e.g., chocolates and toffees.	Removed	0.539
C4	Products at the point of sale help my kids in persuading me to buy as they give chocolates and toffees in exchange for money.	Retained	0.687
C5	My kids gather information about a product to persuade me to purchase it for them.	Retained	0.744
C5	My kids gather information about the place of purchase of products to persuade me to purchase it for them.	Retained	0.686
C5	My kids will demand certain products so that their friends will like them.	Retained	0.827
C5	My kids demand a certain product that their friends possess so my kids feel confident.	Retained	0.835
C5	My kids demand a certain product that their friends possess so that my kids feel satisfied.	Retained	0.826
C5	My kids demand a certain product that their friends possess so my kids feel happy and cool.	Retained	0.787
C6	My child persuades me to buy a product of his/her preference by asking.	Removed	0.204
C6	My child persuades me to buy a product of his/her preference by pleading.	Removed	0.620
C6	My child persuades me to buy a product of his/her preference by bargaining.	Retained	0.747
C6	My child persuades me to buy a product of his/her preference by lying.	Retained	0.759
C6	My child persuades me to buy a product of his/her preference by keeping an innocent face.	Removed	0.578
C6	My child persuades me to buy a product by asking repetitively for the product yet trying not to irritate us.	Retained	0.744
C6	My child persuades me to buy a product by becoming especially affectionate towards us in the hope to get his/her way.	Retained	0.714
C6	My child persuades me to buy a product by providing us with rationale for buying a product.	Removed	0.515
C7	When I buy the product that my child has demanded, it makes him/her happy to possess palatable food items, e.g., pizzas and burgers.	Removed	0.454
C7	When I buy the product that my child has demanded, it makes him/her possessive about delicious food.	Retained	0.652
C7	When I do not buy a product of self/family consumption that my child has demanded, it results in conflict between me and my child.	Retained	0.830
C7	When I do not buy a product of self/family consumption that my child has demanded, it results in aggressive behavior such as showing anger and disobedience.	Retained	0.795
C7	When I do not buy the product of self/family consumption that my child has demanded, it results in dissatisfaction in the child.	Retained	0.832
C7	In case of conflict in the choice of product or brand, we consider the child’s request if the product is of use for the child.	Removed	0.397
C7	My kids never displays aggressive behaviors in public.	Removed	0.287
C7	I do not feel judged by strangers when my kids displays aggressive behaviors in public.	Removed	0.272
C7	I have a feeling that I end up purchasing unhealthy food due to my kid’s persuasion.	Removed	0.502
C7	I feel judged by strangers when my preschooler displays pestering behaviors in public.	Retained	0.563
C8	I am happy that I could engage in shopping activities along with my child/children.	Retained	0.745
C8	I felt satisfied in making purchase decisions with my children and share the experience with my friends and peers.	Retained	0.722
C8	I feel happy that my child is capable of selecting products that match their needs.	Retained	0.671
C8	Many times, I buy a product that my child demands/suggests.	Retained	0.886
C8	I prefer to take my children along with me when I purchase items.	Retained	0.885
C8	The total shopping experience with my children was really exciting.	Retained	0.874

## Appendix B. Construct Measurement Sources

**Table A2:** Sources of the measurement items used for each construct.

Construct	Source of Measure
C1—Parental Engagement	Adapted from the parent–child communication dimension of Consumer Socialization Theory (Moschis & Churchill, 1978; Moschis, 1985) and contextualized for parental engagement in Indian family food-purchase settings
C2—Packaging & Brand	Adapted from Christino et al. (2019) and contextualized for Indian packaged-food branding and packaging cues
C3—Advertising	Adapted from McNeal (1992) and Wakefield and Inman (2003) and contextualized for television advertising exposure among Indian children
C4—In-Store Promotions	Adapted from Haglund and Stenberg (2012) and contextualized for point-of-sale promotional practices in Indian organized retail
C5—Peer Influence	Adapted from Jain and Sharma (2016) and contextualized for peer-influence dynamics among Indian children
C6—Pester Power	Adapted from Nicholls and Cullen (2004) and Kaur and Singh (2006) and contextualized for pester-power tactics observed in Indian family settings
C7—Conflict	Adapted from Buijzen and Valkenburg (2003) and contextualized for parent–child conflict following purchase refusal in Indian households
C8—Co-Shopping	Adapted from the parent–child communication and joint decision-making dimension of Consumer Socialization Theory (Moschis & Churchill, 1978; Ward, 1974) and contextualized for co-shopping experiences in Indian retail environments

Note. This table specifies, for each construct, the published measure from which its items were adapted and the context to which they were adapted. Item-level wording for each construct is reported in Appendix A.
